# A Comparative Study of the Application of Fluorescence Excitation-Emission Matrices Combined with Parallel Factor Analysis and Nonnegative Matrix Factorization in the Analysis of Zn Complexation by Humic Acids

**DOI:** 10.3390/s16101760

**Published:** 2016-10-22

**Authors:** Patrycja Boguta, Piotr M. Pieczywek, Zofia Sokołowska

**Affiliations:** Institute of Agrophysics, Polish Academy of Sciences, Doświadczalna 4, 20-290 Lublin, Poland; p.pieczywek@ipan.lublin.pl (P.M.P.); z.sokolowska@ipan.lublin.pl (Z.S.)

**Keywords:** metal complexation, fluorescence spectroscopy, excitation-emission matrices, decomposition methods in modeling metal complexation, humic acids

## Abstract

The main aim of this study was the application of excitation-emission fluorescence matrices (EEMs) combined with two decomposition methods: parallel factor analysis (PARAFAC) and nonnegative matrix factorization (NMF) to study the interaction mechanisms between humic acids (HAs) and Zn(II) over a wide concentration range (0–50 mg·dm^−3^). The influence of HA properties on Zn(II) complexation was also investigated. Stability constants, quenching degree and complexation capacity were estimated for binding sites found in raw EEM, EEM-PARAFAC and EEM-NMF data using mathematical models. A combination of EEM fluorescence analysis with one of the proposed decomposition methods enabled separation of overlapping binding sites and yielded more accurate calculations of the binding parameters. PARAFAC and NMF processing allowed finding binding sites invisible in a few raw EEM datasets as well as finding totally new maxima attributed to structures of the lowest humification. Decomposed data showed an increase in Zn complexation with an increase in humification, aromaticity and molecular weight of HAs. EEM-PARAFAC analysis also revealed that the most stable compounds were formed by structures containing the highest amounts of nitrogen. The content of oxygen-functional groups did not influence the binding parameters, mainly due to fact of higher competition of metal cation with protons. EEM spectra coupled with NMF and especially PARAFAC processing gave more adequate assessments of interactions as compared to raw EEM data and should be especially recommended for modeling of complexation processes where the fluorescence intensities (FI) changes are weak or where the processes are interfered with by the presence of other fluorophores.

## 1. Introduction

Humic acid (HA) is a complex mixture of degraded organic compounds formed by complex, biochemical processes. HA plays an essential role in the environment by improving the air-water interactions, porosity, viscosity, compaction and buffering properties of soils. HA is also a key source of energy for the activity of microflora and -fauna of soils and take part in the regulation of oxidation-reduction processes [1]. However, the most important functions of HA result from its high reactivity and great sorption capacity, in the range of 500–1200 cmol/kg [2]. HA strongly interacts with metals and as a consequence controls chemical speciation, bioavailability, toxicity, and mobility of heavy metals in the biosphere [3,4,5,6,7].

Zinc (Zn) is an essential micronutrient that in low concentrations is necessary for plant growth and development. However, it should be emphasized that Zn deficiency is a widespread problem. Alloway [8] reports that almost half of the world’s cereals are grown on zinc-deficient soils, which can negatively affect total crop yields. The availability of Zn can be improved by interactions with humic substances. Soluble complexes of HA-Zn(II) increase availability of this metal by the gradual release of this element for plant consumption. The study of interaction between Zn and HA has been a topical and open subject, however this issue is complex and still unclear, mainly due to the fact that speciation of HA-Zn compounds is strongly determined by pH, Zn/HAs concentration and properties of the HA themselves. Some authors emphasize that HA, depending on origin, climate and kind of organic substance, shows high diversity of molecular mass, humification degree and content of functional groups, including oxygen reactive groups and structures containing P, N and S atoms [3,7,9]. Such chemically varied compounds can demonstrate different metal binding affinities, even for the same heavy metal [10].

Excitation-emission fluorescence 3-D matrices (EEMs) are modern, sensitive and non-destructive analyses which provide highly detailed information on structure and chemical processes. Recently, fluorescence spectroscopy has been applied extensively to molecular characterization, differentiation and classification of humic substances as well as to monitor their changes in waste water or during processes of composting, mineralization or humification [3,11,12,13,14]. However, fluorescence detection can also be applied as an effective technique for understanding the mechanisms of intramolecular and intermolecular interactions between metals and humic acid [15,16]. This utility results from the fact that functional groups of HA demonstrate rich fluorescence “fingerprints”, which can be altered by complexing metals [15,17]. EEM spectra reveal the presence of different fluorophores and as a consequence enable the observation of changes in individual structures of HA. However, HA fluorescence is composed of various types of overlapping fluorophores and interpretation of entire EEM data sets may give misleading results, requiring additional processing for improving resolution of the signal [7,18]. One method that enables the decomposition of fluorescence EEM spectra is parallel factor analysis (PARAFAC). Other authors have used it, but mostly for characterization and differentiation of dissolved organic matter [19,20,21]. There are no many studies regarding PARAFAC analysis in studies of zinc complexation by DOM [22,23,24], however they show encouraging and valuable results of using this method. Another decomposition method, non-negative matrix factorization (NMF) has not been previously applied until now to evaluate HA-Zn interactions.

The NMF algorithm has already been used in a broad range of applications such as text mining models [25], face recognition algorithms [26,27], separation of analytes in nuclear magnetic resonance spectroscopy [28], resolution of graph matching problems (Jiang et al. [29]), and source separation in digital audio signals [30]. In spectral analyses the NMF provides blind positive signal separation by decomposition of the data matrix into the product of two meaningful matrices in that way the remaining data maximizes the variance of processed data set. NMF gives the resolution of complex mixtures where no or little prior information is available. The non-negativity constraint ensures that the profiles of pure-components have physical meaning and can be interpreted as concentration profiles and spectra. As a matter of fact both methods (PARAFAC and NMF) belong to a family of decomposition algorithms that rely on a similar concept of data processing. The main difference between both approaches lies in the assumed model of the input data−the NMF algorithm uses a bilinear input data model, while three-way analysis is performed in the case of the PARAFAC method.

On the basis of the above, the main aims of this paper were: (1) application of excitation-emission fluorescence matrices to provide qualitative and quantitative information on the interaction mechanisms between HA and Zn(II) ions, (2) to test two supporting methods: parallel factor analysis (PARAFAC) and nonnegative matrix factorization (NMF) for decomposition of EEM spectra into independent, well-resolved groups of fluorophores, and (3) to investigate the influence of chemical properties of humic acids on the complexation processes with Zn(II) ions at pH 6.00.

## 2. Materials and Methods

### 2.1. HAs Chemical Characterization

HAs were isolated from well-humified A-horizons of five different soils in order to compare Zn complexation with the compounds of varied properties like humification degree, elemental composition or functional group content. Type of soils and sampling location data are presented in Table 1.

Their physicochemical properties were extensively discussed elsewhere [31]. The extraction of HAs was performed according to the procedure recommended by the International Humic Substances Society (IHSS) [32]. Purified HAs were characterized to assess their chemical properties. The nitrogen (N) as well as carbon (C) content, expressed as weight percent, was determined using a CHN 2400 analyzer (Perkin Elmer, MA, USA), and then the atomic ratio of N/C was calculated. The surface charge at pH 6.00 (Q6), which describes the amount of negatively charged functional groups, was estimated from potentiometric titration curves (Titrino 702 SM, Metrohm AG, Herisau, Switzerland) according to Boguta and Sokołowska [1]. The UV-VIS spectral ratio E254/E436 provides information on structure aromaticity, aliphaticity, molecular weight and maturity of organic particles. This parameter was calculated on the basis of absorbance values measured for HA solutions of 40 mg·dm^−1^ in 0.05 M NaHCO_3_ at wavelengths of 254 and 436 nm (UV-VIS spectrometer, Jasco V-520, Jasco, Tokyo, Japan). Each analysis was performed in triplicate and the results averaged. The content of COOH and OH functional groups as well as humification parameters E4/E6 and ΔlogK were determined as described in detail in previous studies [33].

### 2.2. Fluorescence Measurements in HA-Zn(II) Systems

Five stock solutions of HAs were prepared by dissolving of 50 mg of each lyophilized sample in 1 dm^3^ of deionized water. The pH of the solutions was adjusted to 8 by adding small amounts of 0.1 M NaOH. A stock solution of Zn ions (1000 mg·dm^−3^) was obtained by dissolving analytical grade zinc chloride in deionized water. For each HA sample, a series of solutions with increasing Zn concentrations was prepared by mixing of constant volumes (40 cm^−3^) of the sample HA stock solution with appropriate volumes of Zn stock solution and deionized water. Thus, each series contained 50 cm^−3^ solutions with constant HAs concentration of 40 mg·dm^−3^ and an increasing amount of Zn(II) ions: 0, 1, 2, 3, 4, 5, 6, 7, 8, 9, 10, 12, 15, 20, 30, 40 and 50 mg·dm^−3^. The pH of each sample in the series was adjusted to 6.00 ± 0.1. All the samples were left on a magnetic stirrer under N_2_ atmosphere for 24 h in order to avoid interferences from sorption of small amount of CO_2_ from the air. Afterward, the pH was readjusted to 6.00 ± 0.1 and emission-excitation fluorescence 3-D matrices (EEM) were recorded for all prepared HA-Zn series using a F-7000 FL luminescence spectrometer (Hitachi, Tokyo, Japan). Emission and excitation slits were set at 10 nm and scan speed adjusted to 12,000 nm·min^−1^. Samples were excited from 250 to 500 nm in increments of 5 nm, whereas emissions were measured at each excitation wavelength from 300 to 600. All measurements were performed at 25 °C to ensure complexation equilibrium and minimize. The fluorescence spectrometer was calibrated. Fluorescence intensity was calibrated using fluorescence intensity of quinine sulphate (λ_ex_ = 350 nm and λ_em_ = 450 nm. Spectral corrections were performed for the instrument using solution of Rhodamine B [34,35].

### 2.3. Fluorescence Data Pre-Processing

**Figure 1:**
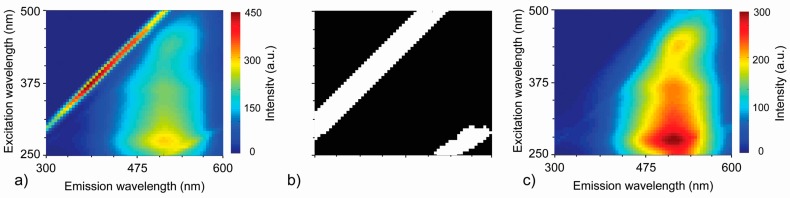
Results of data pre-processing: (**a**) raw EEM map; (**b**) binary mask with Rayleigh peaks marked on white; (**c**) final, processed data.

Scatter signal on EEM spectra is often large and therefore should be deleted before modeling [35]. All fluorescence maps/landscapes were processed via a MATLAB protocol designed to remove first- and second-order Rayleigh peaks (Figure 1). Peaks were removed using simple binary thresholding based on magnitudes of gradients of the fluorescence data. Gradient magnitudes were computed from directional orthogonal gradients, G_x_ and G_y_, with respect to the x-axis and y-axis (defined along the columns and rows of the data matrix, respectively). The threshold level was expressed as the percentage of maximum value of gradient magnitude. Regions of gradient map with values above the threshold value were selected for removal. The binary mask resulting from thresholding operation was dilated using a disk-structuring element with an arbitrarily chosen radius.

Corrupted data indicated by the binary mask was removed by an inpainting procedure where each marked region of original data was filled using an iterative process based on two-dimensional convolution using a Gauss kernel. At the very beginning, selected regions were uniformly filled with values equal to the global average from the original fluorescence data (Figure 1c). The next operation involved smoothing using the Gauss kernel. The part of the smoothed map indicated by binary mask was preserved while the rest of the image was replaced with original data. This process was repeated and at each iteration, the regions of corrupted data were gradually filled in with smoothed data from the neighboring regions.

### 2.4. Fluorescence Data Unmixing

**Figure 2:**
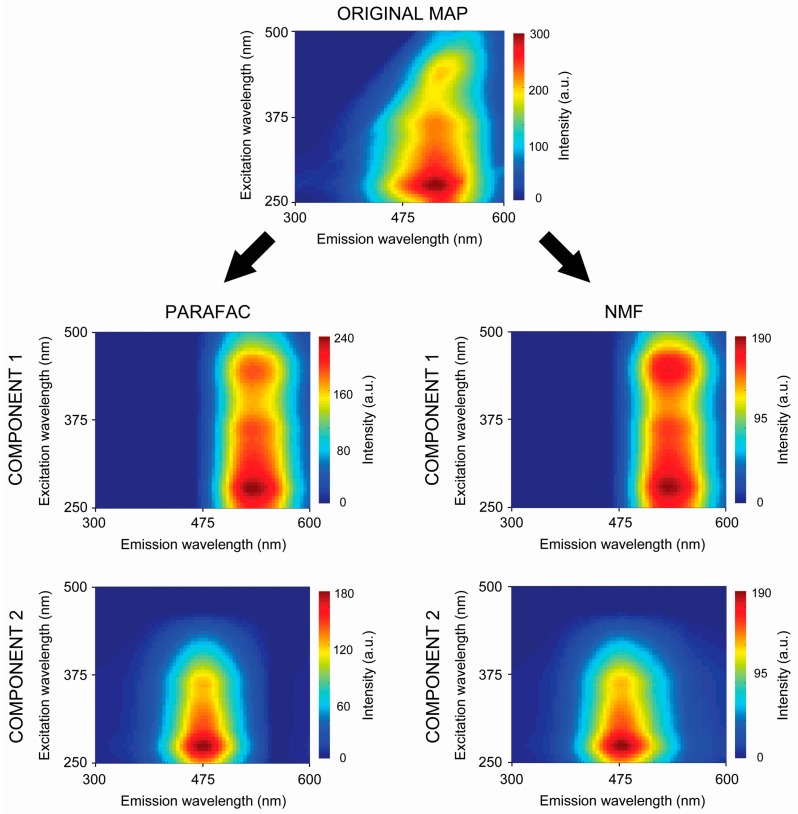
Example results of raw fluorescence data decomposed into two separate components by means of PARAFAC and NMF algorithms.

All fluorescence maps were decomposed into two separate components using the PARAFAC and NMF algorithms. A scheme of the decomposition is shown in Figure 2. Then, local maxima searches were performed using the original pre-processed EEM maps (simply marked as RAW data) and all component maps (designated as PARAFAC and NMF data). Searching was based on the local neighborhood defined by a circle with an arbitrarily chosen radius.

#### 2.4.1. Nonnegative Matrix Factorization (NMF)

The goal of NMF is to decompose an *M* by *N* data matrix ***A*** into the products of two factor matrices ***W*** and ***S*** of reduced size, and can be written as:

(1) A≅WS,

(2) $$A≅\left(\begin{array}{ccc} w_{1,1} & \cdots & w_{1,K}\\ \vdots & \ddots & \vdots \\ w_{M,1} & \cdots & w_{M,K} \end{array}\right)\left(\begin{array}{ccc} s_{1,1} & \cdots & s_{1,N}\\ \vdots & \ddots & \vdots \\ s_{K,1} & \cdots & s_{K,N} \end{array}\right),$$

The rank value *K* describes the largest collection of linearly independent components of the data matrix. For fluorescence data sets, *K* is interpreted as the number of pure chemical components in the sample. In this study, the number of rows *M* corresponds to the number of excitation wavelengths, while the number of columns *N* to the number of emission wavelengths. The resulting ***W*** matrix is interpreted as the matrix of excitation profiles for individual components. Similarly, the ***S*** matrix consists of the emission profiles of components. The multiplication of *K*-th column of ***W*** by corresponding row of ***S*** gives the *M* by *N* fluorescent map of *K*-th component.

Usually NMF algorithms need an estimation of the number of components in the system. In this study, such estimations were provided by the Singular Value Decomposition algorithm (SVD). The rank value *K* was determined by examination of the non-zero singular values of the singular matrix resulting from SVD decomposition of matrix ***A***.

In this study, the factors ***W*** and ***S*** were chosen to minimize the root-mean-squared residual between ***A*** and ***WS*** by means of a multiplicative update algorithm. The algorithm used in this study was developed in MATLAB and is based on build-in implementation of the NMF algorithm.

#### 2.4.2. Parallel Factor Analysis Model (PARAFAC)

A PARAFAC model of a three-way array is given by three loading matrices, ***A***, ***B***, and ***C*** with elements *a_in_*, *b_jn_*, and *c_kn_*. The model was independently proposed by Harshman [36] and by Carroll and Chang [37]. The trilinear model is found to minimize the sum of squares of the residuals, *e_ijk_* in the model:

(3) $$x_{ijk}=\sum_{n=1}^{C}a_{in}b_{jn}c_{kn}+e_{ijk},$$

where *x_ijk_* is the measured value of emission intensity of the *i*-th fraction of the *k*-th sample measured at the *j*-th excitation wavelength; *a_in_*, *b_jn_* and *c_kn_* represent the parameters to estimate; *r_ijk_* are the residuals; and *C* is the number of factors/components extracted.

In this solution each component consists of one score vector and two loading vectors. Using the model it is possible to resolve the data into meaningful components corresponding to individual analytes. The loading matrices ***A***, ***B*** and ***C*** are estimated in a least squares sense. In this study, two-way arrays were processed as input, therefore the third mode of the model was interpreted as a one-dimensional mode. Also, non-negativity constraints were put on the loadings of the different modes. Calculations were performed using the N-way PARAFAC toolbox (version 3.30) for MATLAB R2013A (MathWorks, Natick, MA, USA) [38]. Scheme of the decomposition process is showed in Figure 2.

### 2.5. Calculation of Binding Parameters

Binding parameters were estimated for pre-processed, original RAW data sets as well as for decomposed data (PARAFAC and NMF) at each maximum. Fluorescence intensities (FI, arbitrary units) of the found peaks (designated when possible as α, β, γ or ω), which changed markedly as a function of Zn(II) concentration, were used in the Ryan and Weber quenching model for quantification of complexation process [39]. As a result, the conditional stability constant of HA-Zn(II) complexes (logK), binding capacity of HAs (*C_L_*) and *I_ML_* values (the FI signal at which no more decrease of FI was observed during Zn addition) were estimated by solving equation of the model:

(4) $$I=I_{0}+(I_{ML}-I_{0}\left(\frac{1}{2KC_{L}}\right)(1+KC_{L}+KC_{M}-\sqrt{{\left(1+KC_{L}+KC_{M}\right)}^{2}-4K^{2}C_{L}C_{M}},$$

where *I*_0_ is the fluorescence intensity of HA without addition of Zn and *C_M_* is a metal concentration.

Moreover, quenching degree f was calculated by Equation (5) [40]:

(5) $$f=\frac{I_{0}-I_{ML}}{I_{0}}\times 100.$$

All EEM plots (RAW, PARAFAC and NMF) were graphically elaborated using Surfer software (Golden Software Inc., Golden, CO, USA).

## 3. Results and Discussion

### 3.1. Chemical Properties of Humic Acids

The studied samples exhibited a wide diversity in term of their chemical properties. Results of analyses that might have any effect on the binding processes of Zn(II) ions are presented in Table 1. As noted above, methods for determining the concentration of oxygen-containing functional groups, E4/E6 and ΔlogK ratios, as well as further discussion of these results is presented in more detail in Boguta and Sokołowska [33]. Briefly, the content of COOH groups was the highest for HA3 whereas the total amount of COOH + OH reached the highest value for HA2. These functional groups constitute a major part of all reactive structures in HA particles so their high concentrations indicated, to some extent, a higher reactivity, sorption and buffering capacity of HA2 and HA3 in comparison to the other samples [1]. Results of ΔlogK coefficient showed that HA3 belonged to the samples with the lowest stage of humification (R-type), whereas all remaining HAs demonstrated a weak degree (B-types) of humification [41]. The values of the E4/E6 ratio decreased in the order: HA3 > HA4 > HA2 > HA1 > HA5 which suggested increasing molecular weight, humification degree, and increasing presence of condensed aromatic structures (highest in HA5, lowest in HA3). The highest value of E4/E6 for HA3 indicated much more quantities of aliphatic structures in comparison to the other samples [42]. Results obtained in these studies showed that E4/E6 order was almost duplicated in the E254/E436 ratios which were ordered as follows: HA3 > HA4 > HA2 > HA5 > HA1 revealing the highest content of unsaturated compounds, conjugated quinones and ketones and large molecular size for sample HA1 and the lowest one for HA3. The highest amount of nitrogen was found in the HA1 sample, whereas the lowest content was measured in HA4. High N concentration indicated an early stage of biological transformation and a higher amount of this element in functional groups like amines and amides or in heterocyclic compounds. The nitrogen-containing structures exhibit abilities for improving HA sorption properties due to their donor electron properties [9]. The highest content of C was measured for HA4 while the lowest one for HA2. These amounts influenced atomic N/C ratio which was the highest for HA1 and the lowest for HA4. All range of calculated N/C ratios was typical for soil HAs. This parameter might be considered as indicator of humification. High values showed also lower degree of organic substance transformation [43]. The value of Q6 was the highest for HA1, which indicated the highest concentration of all negatively charged functional groups (not only COOH + OH), however, limited to the structures dissociating below pH 6.00 (the pH of the present experiments on HA-Zn systems). The results showed that this parameter did not correspond with the number of COOH and COOH + OH groups, which indicated a contribution of other functional groups to Q6 values and dissociation of COOH and OH partly above a pH of 6.00.

It should be noted that the process of extraction, purification and drying of HA can have slight effect on chemical and physical properties of HA. However these procedures are necessary when interactions with metals are performed as well as when abilities of HAs from different sources are compared. Modification of the structure can be minimized by using well designed procedures as in case recommended by IHSS [32].

### 3.2. Characterization of Humic Acids Using Fluorescence Spectroscopy

#### 3.2.1. RAW Data

**Figure 3:**
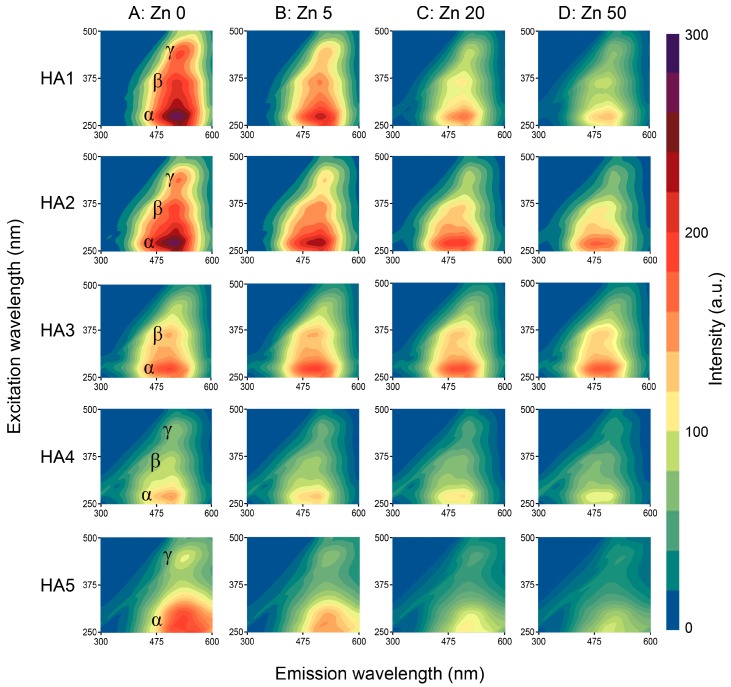
EEM-RAW fluorescence matrices of the studied HAs samples: without Zn (column A) and with example Zn concentrations (columns B, C and D).

Direct analysis of the EEM RAW data showed the presence of two or three local maxima of fluorescence, depending on the HA sample. Results are depicted in Table 2 (ex/em coordinates of the peaks) and in Figure 3 (HAs without Zn ions: column A, rows: 1–5) where the local maxima are marked as α, β and γ. All of the α, β and γ signals corresponded with the presence of various kinds of humic type structures [4,14,44]. The α signals, placed at excitation/emission wavelengths 270–275 nm/490–505 nm were visible in all studied HAs with the highest FI signal for HA1 and HA2, which indicated a higher amount of electron-donating substituents like hydroxyl or methoxy groups for these samples [40]. According to the literature, the α area was related to terrestrial HA derived from lignin [7,45] and might reflect simple structural components of low molecular weight, low content of conjugated and aromatic groups and low humification degree [6,46,47]. The activity of β area was visible at wavelengths 360–365 nm/490–500 nm (partly overlapped by the α peak) with the strongest signal for HA1 and the weakest one for HA4. Fluorescence of this maximum could be attributed to the presence of moderately humified structures, simple phenols, coumarins, alkaloids, semiquinones and hydroquinones [47,48]. Only HA5 did not demonstrate a fluorescence maximum at β site. The γ area was applied to maximum placed at 435–445 nm/505–525 nm which could be ascribed to the presence of unsaturated bonds, high molecular weight and high humification degree as well as to linearly-condensed aromatic rings formed mainly from lignin oxidation [11,16,46]. These sites were clearly seen for all HAs except for HA3. The lowest FI of this site for HA3 and HA4 could indicate a great concentration of electron-withdrawing groups like carbonyls [16,40].

#### 3.2.2. PARAFAC and NMF Processing

**Figure 4:**
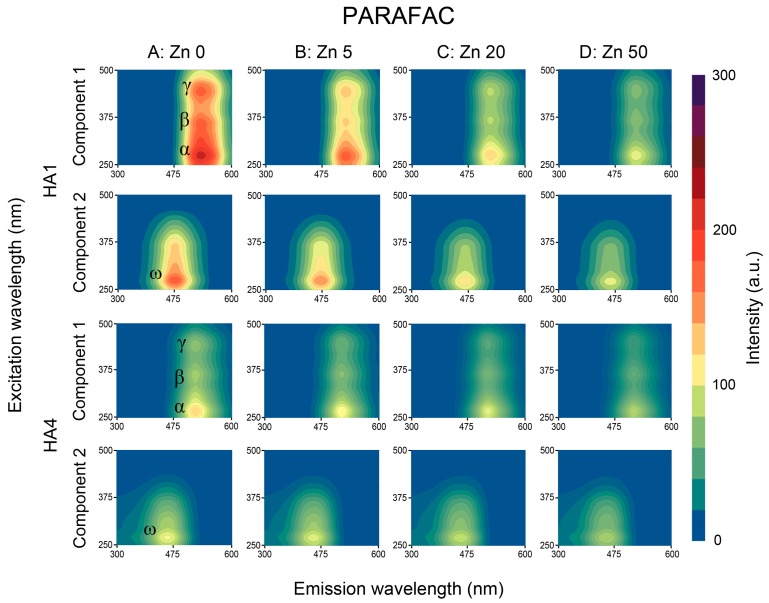
EEM-PARAFAC fluorescence matrices of HA1 and HA4 samples: Without Zn (column A) and with exemplary Zn concentrations: 5, 20 and 50 mg·dm^−3^ (columns B, C and D). For each HA: upper plot: Component 1, lower: Component 2.

**Figure 5:**
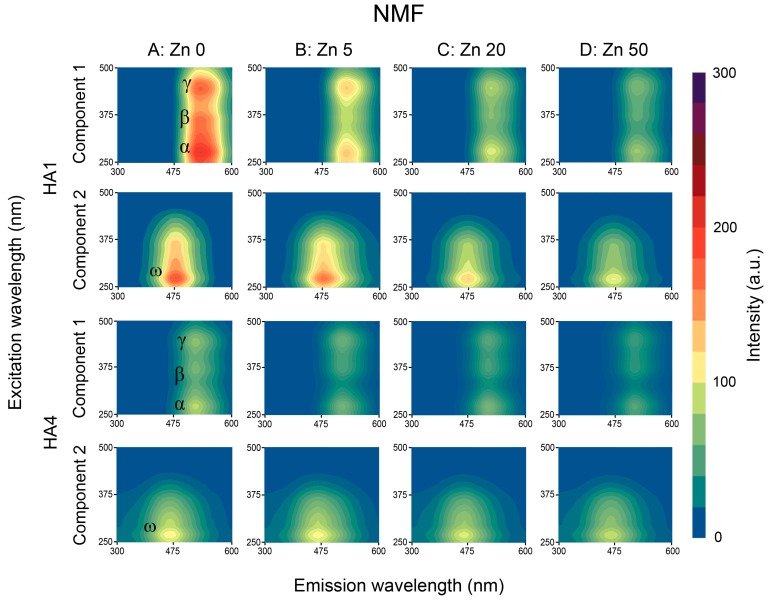
EEM-NMF fluorescence matrices of HA1 and HA4 samples: without Zn (column A) and with exemplary Zn concentrations: 5, 20 and 50 mg·dm^−3^ (columns B, C and D). For each HA: Upper plot: Component 1, lower: Component 2.

Decomposition of EEM matrices to the two components using PARAFAC and NMF algorithms revealed the presence of additional local maxima as well as improved resolution of the peaks found in analysis of RAW data. Two-component analysis was also recognized in previous studies by Mounier et al. [49] and Santos et al. [45] as sufficient for separation of binding sites. Example EEM-PARAFAC and EEM-NMF matrices for the HA1 (the strongest FI) and HA4 (the lowest FI) are depicted in Figure 4 and Figure 5 (column A).

Maxima of component 1 corresponded in terms of excitation wavelength λ_ex_ with the peaks found for RAW data in the α, β and γ areas (Figure 3: Column A), whereas both the PARAFAC and NMF decomposition revealed slight changes in the λ_em_ of the α, β and γ areas toward longer wavelengths. The PARAFAC and NMF decomposition processes uncovered a noticeable γ peak for HA3, which was invisible during the RAW data evaluation.

Component 2 obtained via PARAFAC and NMF processing revealed the presence of a whole new maximum (marked as ω) of low intensity, located in the short wavelengths: 260–275 nm/430–470 nm (see Table 2—Detailed coordinates). Fluorophores similar to ω were also confirmed by Mounier et al. [49] in two-component decomposition. This activity could be attributed to some contents of carbohydrates and electron-withdrawing carboxylic functional groups, which may be responsible of the small fluorescence intensity [16,46] as well as to structures of low humification and molecular weight, with natures more similar to derivatives of amino acids and other heterocyclic compounds [47].

Both γ (for HA3) as well as ω peaks (for all HAs) were concealed in the original EEM spectra, which was likely caused by overlapping signals of neighboring fluorophores. PARAFAC and NMF algorithms enabled better separation of the above maxima for their further elaboration. The total number of local maxima found via the PARAFAC method was the same as for NMF and the differences in their locations were negligible. FI values of the peaks in component 1 were higher for PARAFAC than NMF but the reverse for component 2.

### 3.3. Quantification of Zn Complexation by Humic Acids

#### 3.3.1. RAW Data

The addition of increasing concentration of Zn(II) ions caused a gradual quench of fluorescence in the areas marked as α, β and γ, which indicated the presence of metal complexation by HAs particles [6,27,40]. Example EEM-RAW spectra displaying the changes of fluorescence intensities of the above peaks under the influence of Zn are shown in Figure 3.

It is notable that the increasing Zn concentration also caused an apparent shift of the λ_em_ of the maximum to shorter wavelengths (blue shift), which could result from: (1) more localization of the π-electron system, (2) branching of the aromatic system [50], (3) a decrease in conjugated double bonds [47], or (4) the presence of a few groups of fluorophores in one area with various affinity to Zn(II) binding. In this case, the structures of the area active at longer wavelengths could be more strongly complexed than the structures active at shorter λ_em_, therefore the maximum of the entire area could be shifted to shorter wavelengths. Similar suppositions were also reported by Hernández et al. [6] and Plaza et al. [16] in studies of copper interactions with humic acids. This process was evidently seen for α binding sites. In turn, the addition of Zn(II) ions did not cause significant changes in λ_ex_ of the analyzed fluorescence maxima.

**Figure 6:**
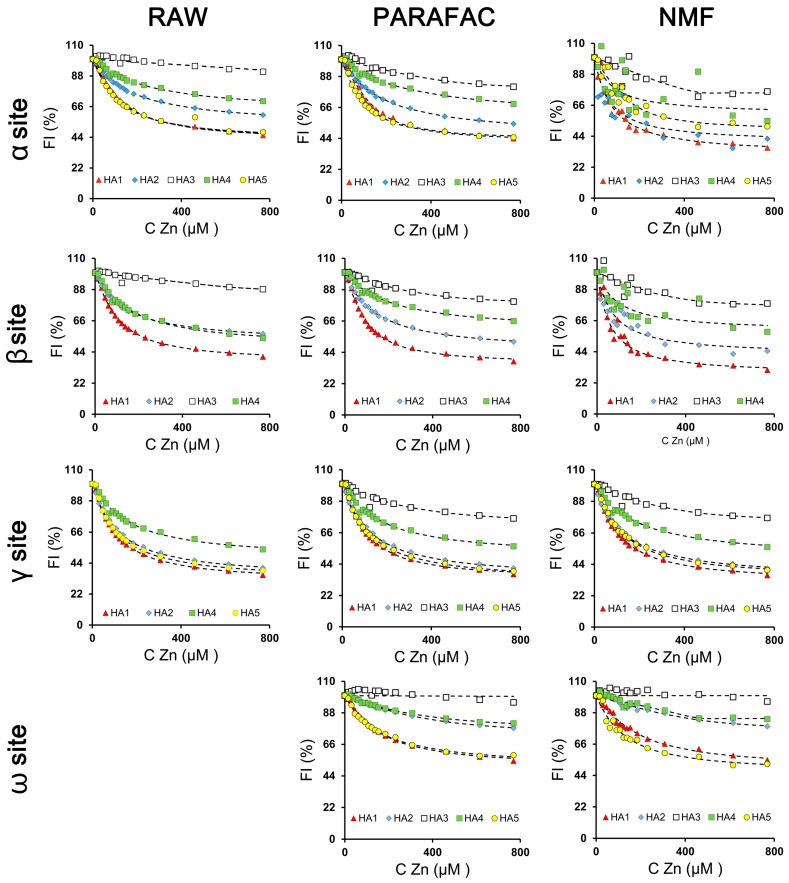
Quenching profiles of HAs fluorescence at α, β, γ and ω maxima of RAW, PARAFAC and NMF data under increasing Zn concentrations. Symbols are denoted for experimental points, solid lines—for model curves.

More detailed information on the mechanism of Zn(II) binding was obtained by fitting experimental RAW data, i.e., changes of FI values at α, β and γ sites, under increasing Zn(II) concentrations, to the mathematical, quenching model of Ryan and Weber which enabled to calculate binding parameters [39]. These fits are presented in Figure 6 as percent changes (I/I_0_ × 100), where I and I_0_ are the fluorescence intensities with and without Zn(II), respectively. Binding parameters estimated from the model are displayed in Table 2.

Results showed that the decrease in FI was the most intensive at the lowest Zn(II) concentrations. This trend likely resulted from the presence of a significant amount of free, available reactive sites on the HA ligands. Metal binding was additionally facilitated at pH 6.00 due to a high degree of functional group dissociation and in the consequence due to a high value of the negative charge (Q6) on the HA surface, which made it easier for positive cations to be attracted to the coordination sphere [1,51]. The decrease of FI was progressively weaker and weaker with increasing Zn dose, until a point above which further metal addition did not cause a significant drop of the FI value, which indicated saturation of available reactive sites by metal ions [39]. It should be noted that the fluorescence signal was not completely quenched in any case even at the highest Zn concentrations. The values of residual fluorescence (IML) calculated from the Ryan and Weber model are presented in Table 2 (α, β, γ—RAW data).

Plaza et al. [16] have reported that uncompleted quenching could result from some steric effects occuring at partly complexed ligands during complexation. Functional groups that bind Zn(II) ions could weaken reactivity of neighboring structures. Incomplete saturation of the functional groups could also be related with spatial conformation of the structures of HA particles. The structures could be partly presented in form of coils hampering access of metal to ligands. It is known that linear and spatial development of the polymeric HA structure increases with increasing pH and reaches a maximum in strongly alkaline conditions [1]. Dissociation degree is the highest at these alkaline pHs and the generated negative surface charge is able to repulse particular chains and prevents spherical structure formation. These studies were conducted at pH 6.00, at which point functional groups of weakly acidic nature like carboxylic or phenolic are not dissociated completely, which can be a reason for partial aggregation of the particles and hampered metal complexation. This supposition was in agreement with the Q6 values, which were lower than value of COOH + OH and lower even in most cases than the content of COOH. The influence of pH on metal binding was also confirmed in previous studies [51,52], which showed an increased proton-binding competition at lower pHs.

The highest relative drop of FI (from I_0_ to I_ML_), expressed as f was observed for all HAs at γ binding sites, which reflected a preferential binding of Zn(II) ions by strongly humified structures with high molecular weight and condensation of aromatic units. The lowest f values were determined for α areas, which indicated a lower contribution of weakly humified and low molecular weight structures in the process of Zn(II) complexation. Considering the studied HAs, the highest drop of FI was observed for HA1 and HA5, whereas the lowest was for HA3, which suggested these had the strongest and the weakest affinities of Zn(II) ions, respectively.

Values of complexation capacity (C_L_) estimated from the model on the basis of RAW data demonstrated wide diversity within the individual fluorescence area as well as in the group of all studied HAs. At the α and γ maxima, the C_L_ was the highest for HA1, whereas at β sites, the C_L_ was highest for HA3. However, it should be emphasized that the C_L_ parameter showed strong sensitivity even to slight deviations of experimental points from the model curve. In our opinion, the parameter that was much more stable for assessment of complexing degree was the f index described in the previous paragraph.

The stability of HA-Zn complexes (logK) calculated from the model at the α and γ binding sites was the highest for HA1, whereas at β sites the stability was highest for HA3. According to the literature, significant stability of HA-Zn binding can suggest the forming of chelates with participation of aromatic rings, highly humified structural units as well as structures containing nitrogen atoms as an electron donor greater than oxygen donor atoms [9,16]. On the other hand, the studies of Garcia-Gil et al. [5] report that low values of logK could be ascribed to stronger aliphatic character and small degree of aromatic polycondensation and humification.

A very good fit of the model to the experimental data (Table 2) was confirmed by its high correlation coefficient (R > 0.92). However the model was not applicable for RAW data at α-sites of HA3. This was probably due to very low changes of fluorescence in α area under increasing Zn doses.

#### 3.3.2. PARAFAC and NMF Processing

Fluorescence local maxima found by means of PARAFAC and NMF processing (α, β, γ in component 1, and new maximum ω in component 2) were quenched with increase in Zn ions concentrations. This process differed for studied HAs as well as for analyzed fluorescent peaks, which was typical for the different origin, properties and components of HAs [7,12,18]. FI changes of decomposed EEM spectra in components 1 and 2 under example Zn doses are shown for PARAFAC and NMF algorithms in Figure 4 and Figure 5. The changes in FI were more pronounced on EEM spectra for the datasets decomposed by PARAFAC and NMF (better resolving of the peaks) than in case of RAW data.

The experimental points from the PARAFAC and NMF methods were applied to the quenching model for quantification of complexation process. High values of R coefficients (Table 2) confirmed a good fit of the obtained models, which showed also similar results when compared with the RAW data. In more detail, for peaks in α and β areas fits were good for RAW and PARAFAC data, showing slightly lower performance for NMF data. In the case of ω and γ fluorescence sites R coefficients were the highest and on similar level for RAW, PARAFAC and NMF data, which indicated the best reliability of the model was for both processing methods in case of ω and γ structures.

Trends in the modeled PARAFAC and NMF data were similar to the RAW data (Figure 6). Such that, the most intensive drop of the FI signal took place at first in the lowest metal concentrations, which was evidence of extensive complexation processes. An Increase in Zn concentration caused FI to decrease until a plateau was reached, indicating saturation of the HA structure by metal ions.

It was interesting that both PARAFAC and NMF decomposition of the experimental EEM data enabled the fitting o model curves to the HA3 data at the α site. The model was not applicable for RAW data of this sample at the α area, due to very low changes of FI under increasing concentrations of Zn. Moreover, as was described in Section 3.3.1, the maximum at the α site of the RAW data was probably due to fluorophores of different kinds. Part of these were complexed and their fluorescence was quenched whereas others remained without any changes, which could be revealed in RAW data as changes in the maximum location of whole area and in consequence could hamper application of the model. Decomposition of the data enabled the separation of neighboring maxima and analysis of the changes only for fluorophores taking part in Zn complexing. Furthermore, the decomposition of the HA3 (α site) data remolded the rectilinear character of FI changes into nonlinear ones with a visible point of inflection, which allowed the use of the model to quantify Zn binding (see Figure 6). These results showed that NMF and especially PARAFAC processing (higher R coefficients) can be recommended for modeling of complexation processes of weak intensity, where the changes of FI are too slight or for processes that are interfered with by the presence of other fluorophores.

PARAFAC and NMF processing also enabled separation of the maximum for HA3 at the γ-area, which was impossible in the analysis of the RAW data. Experimental points of this area after PARAFAC and NMF treatment fit well to the model with high R-values of 0.96 for these two methods. This was possible due to better resolving of the fluorescent sites neighboring γ and β. In analysis of Zn complexation in the RAW data, fluorescence at β areas was much more intensive than at γ, and probably part of the signal from β sites could interfere with analysis of changes at γ sites. PARAFAC and NMF decomposition eliminated this problem.

A positive feature of the application of the PARAFAC and NMF algorithms was the opportunity of analysis of Zn interactions in the newly found ω area. This fluorescent site was hidden in the RAW data. This peak was confirmed by Santos et al. [45] as being partly covered by neighboring fluorophores. The ω structures attributed to very low humification and groups of low molecular weight [47], demonstrated a tendency for complexation of Zn ions, which was visible as quenching of the FI with increasing metal concentration. The drop in FI with increasing Zn concentration for each HA at the ω area was lower than at α, β and γ sites, which could indicate a lower importance of ω structures with simple units, low molecular weight and aromaticity in comparison to highly humified and condensed structures with large molecular mass. The ω data fit very well to the model despite the low FI of this area as well as weak changes when Zn ions were increased. The only case in these studies when model curve could not be fit to experimental data of ω sites after treatment by the PARAFAC and NMF algorithms was related to the HA3 sample. However, it should be emphasized that Zn complexation degree expressed by fluorescence changes was negligible in this case.

The stability constants (logK) of individual HAs at α, β and γ areas estimated using the PARAFAC and NMF methods showed some differences when compared to logK values calculated from RAW data. The lowest discrepancies occurred between the values of γ sites. Higher differences were found between logK at α and β, which indicated that the decomposition process could affect more experimental data of above areas in comparison to γ fluorescent sites. This probably was due to partial interference of α with β and α with ω fluorescent areas. Decomposition of the EEM matrices caused better separation of these maxima, which could be translated into higher differences in logK values in comparison to the RAW data. Therefore, it might be assumed that logK values from NMF and especially PARAFAC processing (higher R indices) had higher accuracy than from RAW data. Despite these differences, the highest stability constants for both RAW, PARAFAC and NMF calculations were demonstrated by HA1, which was due to its greater nitrogen content; N-containing functional groups are considered to be ligands with strong affinity to metals [11,53]. HA1 data revealed also the highest value of Q6 and the lowest E245/E436 ratio, which could also substantially determine the stability of these complexes. This reflected that it has a greater concentration of negatively charged functional groups and the highest content of unsaturated compounds, such as conjugated quinones and ketones, which might be of key importance in Zn binding. It should be stressed that all studied HAs showed the lower logK values for ω structures than other areas, which pointed out weaker complexing properties of the lowest humified and low-molecular groups and, in the consequences, formation of clearly weaker complexes.

The degree of quenching (f) calculated for PARAFAC and NMF data was similar to the values obtained from RAW data and showed the highest values for HA1 and HA5 at each fluorescent area. These samples were characterized by the highest concentration of nitrogen (N) and value of negative charge at pH 6.00 (Q6) as well as by the greatest humification degree, molecular weight and content of unsaturated structures (expressed by the lowest E4/E6 and E254/E436), which could have a dominant influence on the amount of bounded Zn ions. According to He et al. [40] large quenching degrees can also be associated with the presence of electron-donating groups. Comparing the particular fluorescent areas of PARAFAC and NMF data, it might be noted that the lowest degree of quenching by Zn(II) was attributed to ω binding sites. This insight together with low logK confirmed weak affinity of ω structures to Zn(II) ions.

### 3.4. Summary of the PARAFAC and NMF Performance

In this study two supporting methods—parallel factor analysis (PARAFAC) and nonnegative matrix factorization (NMF)—were applied for decomposition of the EEM spectra into independent, well-resolved groups of fluorophores. The EEM data analysis showed that NMF algorithm has a potential use as an alternative method to the PARAFAC decomposition. In terms of the qualitative performance of decomposition the NMF algorithm showed comparable results with the PARAFAC—by constraining the model to two components we obtained the same number of peaks with similar excitation-emission coordinates. Both sets of the EEM decomposed maps shown in Figure 4 and Figure 5 reveal similar features for the two components, with only slight different intensities for peaks from sites β and ω. This lower performance of the NMF algorithm was reported in terms of quantitative representation of individual fluorescence sources, especially in terms of overlapping of signals. This is clearly visible when we compare quenching profiles for α and ω sites obtained by means of the PARAFAC and NMF. The peak at the ω site was previously not visible at raw EEM maps. It’s presence was successfully revealed as a separate signal belonging to the second component by both applied methods. However due to presence of neighboring peak at the α site the unmixing process performed by means of the NMF algorithm in some cases led to suboptimal solution. The less stable unmixing process, resulted in a wider spread of data points used to build quenching profiles. Better performance of the NMF algorithm can be achieved by tightening the termination tolerance on change in size of the residual or increase in the maximum number of iterations. Another possibility is to change the algorithm which minimized the root-mean-squared residual. The applied multiplicative algorithm is more sensitive to initial values of the resolved system (which by default were randomly chosen), while the alternating least squares algorithm is known to converge faster and more consistently.

### 3.5. Influence of Chemical Properties of HAs on the Complexation Process of Zinc(II) Ions

Statistical analysis confirmed a significant influence of the chemical properties of HAs on the process of Zn(II) complexation at pH 6.00. Correlation coefficients found between selected properties and parameters describing complexation, i.e., logK and f values at α, β, γ and ω are shown in Table 3.

Degree of quenching (f) did not show in any case a significant relationship to the content of COOH and COOH + OH functional groups. This indicated that oxygen-containing groups did not exhibit a dominant influence on the amount of bounded metal. The reason could be found in partially protonated COOH and OH groups at pH 6, at which proton-binding competition can hamper interaction with the total number of COOH and COOH + OH functional groups. Previous studies have shown that full dissociation takes place at higher pHs, even at alkaline conditions in case of OH groups [51,52]. On the other hand, the f parameter displayed negative correlations in the α, β and γ areas with E4/E6, E254/E436, and ΔlogK. A number of these significant correlations with the f-parameters was higher in PARAFAC and NMF data than with parameters estimated from RAW data. The negative direction of the these relationships confirmed a high affinity of Zn ions to HAs characterized by high humification, molecular weight, aromaticity and great content of unsaturated structures, which was also reported by Plaza et al. [16].

The stability of HA-Zn(II) complexes was determined by the nitrogen content, which was confirmed by positive correlations of logK with N for structures at α sites of RAW data, as well as at α and β binding sites in the PARAFAC data (Table 3, data with asterisks). These results can be explained by strong electron donor properties of nitrogen atom, stronger even than oxygen atoms [9,53]. Moreover, the presence of nitrogen in form of side chain functional groups including mainly moieties of amines, amides, amino sugars or peptides involved in linkages between the aromatic groups favors formation of chelates in which metal is built into the ring [53,54,55]. Such types of compounds reveal exceptionally high stability. This fact may justify statistical significance of the relationship with N and simultaneously its absence in relationship to content of COOH and COOH + OH. Above relationship between stability of complexes and nitrogen was also confirmed by positive correlations with N/C atomic ratio which can be considered as the relative abundance of N. Directions and values of R-coefficients were similar to R values of correlations with nitrogen however one more statistically significant correlation was found for the NMF data in case of N/C ratio.

The stability constants of HA-Zn(II) complexes of γ structures significantly increased with the decrease in E4/E6 and E254/E436 ratios, which indicated formation of the strongest bindings with the highly humified structures of high molecular weight, rich in unsaturated bonds and linearly-condensed aromatic rings. However, it should be noted that these correlations were not found in evaluation of the data from PARAFAC and NMF processing. Plaza et al. [15] reported that logK was negatively correlated with the content of COOH and total number of COOH and OH groups in case of interaction with copper. Lack of this dependency in our studies may suggest a high selectivity of complexation process by HA. Stability of the complexes of ω structures did not show significant relationships with any analyzed chemical properties of HAs, probably due to fact of low importance of these structures in Zn binding.

## 4. Conclusions

To summarize, results obtained in these studies showed that EEM fluorescence matrices combined with PARAFAC or NMF processing as well as with data modeling can be considered a promising approach to provide better insight into the mechanisms of metal complexation by specific structural units of HAs.

Elaboration of RAW data enabled finding three and in some cases only two fluorescent peaks, which corresponded with binding sites of HAs. Maxima of the lowest intensities for the moderately and strongly humified structures of β and γ areas were in some samples invisible because of overlapping by neighboring stronger signals. The model was not applied in such cases and the binding parameters could not be estimated. Combination of EEM fluorescence analysis with one of the proposed decomposition methods, PARAFAC or NMF, enabled better separation of the overlapped binding sites and yielded more accurate calculations of the binding parameters. The results showed that PARAFAC and NMF processing worked even for low intensity data and allowed finding binding sites for β and γ areas, which was impossible in the analysis of a few RAW data sets. Both PARAFAC and NMF processing also enabled the observation of a new local maximum (ω), which was attributed to the very simple structures of the lowest humification and molecular weight with the chemical nature similar to derivatives of amino acids and other heterocyclic compounds. Data obtained using PARAFAC and NMF techniques fit very well with the applied complexing model at the γ and ω areas and slightly better for PARAFAC than NMF with the data from the α and β peaks.

Detection of fluorescence changes due to Zn addition demonstrated high sensitivity and this, in turn, provided the possibility for observation of influence of various HAs properties on the Zn complexation process. EEM combined with PARAFAC and NMF methods gave a higher number of statistically significant relationships of the above properties with binding parameters in comparison to RAW data, indicating better preparation of the data for further calculations. As a result, EEM-PARAFAC and EEM-NMF methods showed an increase in quenching degree of fluorescence under increasing concentrations of Zn ions with increase in humification, aromaticity, molecular weight and amount of unsaturated bonds of HAs. EEM-PARAFAC analysis revealed also that the most stable compounds were formed by the HAs containing the highest amounts of nitrogen, which was especially seen for the α and β binding sites. The content of COOH and COOH + OH functional groups did not significantly influence the binding parameters, mainly due to partial protonation of these groups and higher competition of metal cation with protons. The area designated as ω was not clearly affected by analyzed properties of HAs, probably due to its low importance in Zn(II) binding processes. In our opinion, decomposition of EEM spectra by PARAFAC or NMF can be recommended for modeling of complexation processes of weak intensity, where the changes of FI are slight or for processes that can be interfered with by the presence of other fluorophores. PARAFAC and NMF processing coupled with EEM spectra gave more reliable and adequate assessment of HA-Zn interactions as compared to RAW, mainly due to possibility of identifying and quantifying contributions of the each unique structural area associated with HAs.

## Figures and Tables

**Table 1:** Chemical properties of the studied HA samples (in part previously reported in Boguta and Sokołowska [33]).

HA No.	Raw Soil Material	Soil location	N (%)	C (%)	N/C	COOH (cmol·kg^−1^)	COOH + OH (cmol·kg^−1^)	Q6 (cmol·kg^−1^)	E4/E6	E254/E436	∆logK
HA1	*Haplic Fluvisol* (Alluvial soil)	51°09′ N/22°59 ′E	5.11	54.81	0.080	321	650	284	5.35	5.00	0.70
HA2	*Haplic Cambisol* (Brown soil)	50°32′ N/24°01′ E	3.70	51.24	0.062	411	741	266	5.69	5.59	0.67
HA3	*Mollic Gleysol* (Black Earth)	50°22′ N/23°39′ E	4.30	53.28	0.069	424	670	254	7.08	8.32	0.89
HA4	*Stagnic Luvisol* (Grey-brown soil)	50°38′ N/22°41′ E	3.35	58.97	0.049	260	499	261	6.17	6.48	0.75
HA5	*Haplic Chernozem* (Chernozem)	51°23′ N/22°35′ E	4.69	56.1	0.072	196	454	278	4.8	5.28	0.65

**Table 2:** Binding parameters calculated for α, β, γ and ω fluorescence maxima of RAW, PARAFAC and NMF data under increasing Zn concentrations.

	**α-Site RAW**						**α-Site PARAFAC**						**α-Site NMF**					
**HAs No.**	**EEWP (nm/nm)**	**I_ML_ a.u**	**logK**	**R**	**f %**	**C_L_ (mmol/g)**	**EEWP (nm/nm)**	**I_ML_ a.u**	**logK**	**R**	**f %**	**C_L_ (mmol/g)**	**EEWP (nm/nm)**	**I_ML_ a.u**	**logK**	**R**	**f %**	**C_L_ (mmol/g)**
HA1	275/500	112	4.11	1.00	60	2.44	275/520	91	4.41	0.99	58	3.85	280/515	62	4.14	0.95	69	0.25
HA2	270/495	139	3.76	1.00	49	0.38	275/510	92	3.75	1.00	56	0.44	505/275	73	4.22	0.63	61	0.25
HA3	275/490	n/a					270/515	103	4.32	0.87	22	12.5	270/515	71	5.91	0.67	25	10.5
HA4	270/490	86	3.71	0.99	38	2.17	270/505	65	3.52	0.98	43	0.60	275/505	48	4.16	0.34	40	0.41
HA5	270/505	182	4.07	0.99	59	1.44	265/525	184	4.19	1.00	60	1.63	280/525	127	4.37	0.91	52	3.89
	**β-Site RAW**						**β-Site PARAFAC**						**β-Site NMF**					
**HAs No.**	**EEWP (nm/nm)**	**I_ML_ a.u**	**logK**	**R**	**f %**	**C_L_ (mmol/g)**	**EEWP (nm/nm)**	**I_ML_**	**logK**	**R**	**f %**	**C_L_ (mmol/g)**	**EEWP (nm/nm)**	**I_ML_**	**logK**	**R**	**f %**	**C_L_ (mmol/g)**
HA1	360/500	75	4.15	1.00	64	1.57	360/520	60	4.31	1.00	65	2.05	360/515	45	4.29	0.96	72	0.25
HA2	365/495	94	3.88	1.00	50	0.25	365/510	70	3.88	1.00	56	0.25	365/505	61	4.17	0.85	58	0.25
HA3	365/490	114	5.08	0.92	13	17.8	365/515	77	4.02	0.96	24	8.00	370/515	59	4.36	0.76	25	8.48
HA4	360/490	32	3.87	0.99	53	1.40	365/505	43	3.73	0.99	42	0.86	370/505	34	4.06	0.61	42	0.25
HA5	n/d						n/d						n/d					
	**γ-Site RAW**						**γ-Site PARAFAC**						**γ-Site NMF**					
**HAs No.**	**EEWP (nm/nm)**	**I_ML_ a.u**	**logK**	**R**	**f %**	**C_L_ (mmol/g)**	**EEWP (nm/nm)**	**I_ML_ a.u**	**logK**	**R**	**f %**	**C_L_ (mmol/g)**	**EEWP (nm/nm)**	**I_ML_ a.u**	**logK**	**R**	**f %**	**C_L_ (mmol/g)**
HA1	435/510	56	4.13	1.00	70	1.50	445/520	56	4.22	1.00	67	2.33	445/515	51	4.10	1.00	69	1.38
HA2	435/505	53	3.97	1.00	67	0.62	440/510	51	3.96	1.00	67	0.68	440/505	50	3.93	1.00	67	0.65
HA3	n/d						430/515	56	3.77	0.96	30	4.87	435/515	56	4.10	0.96	27	8.10
HA4	435/505	32	3.87	0.99	53	1.40	440/505	33	3.83	0.99	52	1.29	440/505	31	3.76	0.99	53	0.71
HA5	445/525	29	4.03	1.00	69	0.85	450/525	27	4.00	1.00	69	0.85	455/525	28	4.07	1.00	66	1.70
	**ω-Site RAW**						**ω-Site PARAFAC**						**ω-Site NMF**					
**HAs No.**	**EEWP (nm/nm)**	**I_ML_ a.u**	**logK**	**R**	**f %**	**C_L_ (mmol/g)**	**EEWP (nm/nm)**	**I_ML_ a.u**	**logK**	**R**	**f %**	**C_L_ (mmol/g)**	**EEWP (nm/nm)**	**I_ML_ a.u**	**logK**	**R**	**f %**	**C_L_ (mmol/g)**
HA1	n.d.						275/450	85	3.97	1.00	51	2.41	275/450	85	3.92	0.99	52	2.87
HA2	n.d.						275/435	134	3.79	0.99	29	7.97	270/440	145	4.09	0.93	26	11.02
HA3	n.d.						275/440	n/a					275/445	n/a				
HA4	n.d.						270/430	71	3.77	0.99	25	6.77	270/440	84	6.88	0.92	16	11.71
HA5	n.d.						260/455	44	3.84	0.99	51	0.59	260/470	48	4.01	0.96	55	1.29

n/a: not applicable, model cannot be applied; n/d: no data, fluorescence maximum was not found.

**Table 3:** Correlation analysis of the relationships between binding parameters of HA-Zn(II) complexes at pH 6.00 and selected chemical properties of HAs.

	**EEM-RAW**						
	**N**	**N/C**	**COOH**	**COOH + OH**	**E4/E6**	**E254/E436**	**∆logK**
**α-area**							
f	0.91	0.97	−0.12	0.08	−0.91	−0.98 *	−0.74
K	0.97 *	0.93 *	−0.39	−0.19	−0.85	−0.86	−0.50
**β-area**							
f	0.09	0.00	−0.65	−0.21	−0.94	−0.95 *	−0.90
K	0.38	0.41	0.57	0.23	0.78	0.80	0.90
**γ-area**							
f	0.22	0.24	−0.48	−0.15	−0.94 *	−1.00 *	−0.97 *
K	0.56	0.55	−0.38	−0.03	−0.88 *	−0.95 *	−0.83
**α-area**							
f	0.22	0.24	−0.51	−0.19	−0.96 *	−0.99 *	−0.99 *
K	0.93 *	0.94 *	0.11	0.15	−0.09	0.02	0.20
**β-area**							
f	0.31	0.33	−0.28	0.22	−0.99 *	−0.99 *	−0.93
K	0.99 *	0.98 *	0.17	0.39	−0.32	−0.29	−0.03
**γ-area**							
f	0.21	0.24	−0.47	−0.15	−0.95 *	−0.99 *	−0.99 *
K	0.69	0.67	−0.25	0.08	−0.74	−0.84	−0.65
**ω-area**							
f	0.95 *	0.92 *	−0.41	−0.21	−0.90	−0.87	−0.56
K	0.94	0.87	0.00	0.19	−0.46	−0.78	−0.13
	**EEM-NMF**						
	**N**	**N/C**	**COOH**	**COOH + OH**	**E4/E6**	**E254/E436**	**∆logK**
**α-area**							
f	0.41	0.45	−0.33	0.02	−0.89 *	−0.97 *	−0.90 *
K	0.06	0.12	0.59	0.32	0.81	0.91 *	0.91 *
**β-area**							
f	0.41	0.42	−0.26	0.24	−0.98 *	−0.98 *	−0.88
K	0.75	0.83	0.65	0.56	0.33	0.36	0.53
**γ-area**							
f	0.19	0.20	−0.45	−0.12	−0.91 *	−0.99 *	−0.97 *
K	0.87	0.95 *	0.18	0.24	−0.14	−0.01	0.12
**ω-area**							
f	0.93	0.92	−0.38	−0.18	−0.93	−0.89	−0.63
K	−0.67	−0.86	−0.25	−0.42	0.78	0.94	0.87

* significance at the 0.05 level.
